# Application of Magnetic-Assisted Polishing Using Metal-Bonded Grinding Wheels for Machining Silicon Nitride Ball Bearings

**DOI:** 10.3390/ma18030677

**Published:** 2025-02-03

**Authors:** Su-Yeon Han, Seung-Min Lee, Ha-Neul Kim, Jae-Woong Ko, Tae-Soo Kwak

**Affiliations:** 1 Department of Mechanical and Aerospace Engineering, Gyeongsang National University, Jinju 52725, Republic of Korea; shadow1009@naver.com; 2 Department of Mechanical Engineering, Gyeongsang National University, Jinju 52725, Republic of Korea; tmdaks7450@naver.com; 3 Nano Materials Research Division, Korea Institute of Material Science, Changwon-si 51508, Republic of Korea; skykim@kims.re.kr (H.-N.K.); kjw1572@kims.re.kr (J.-W.K.); 4 Department of Aerospace Engineering, Gyeongsang National University, Jinju 52725, Republic of Korea

**Keywords:** silicon nitride, ball bearings, magnetic-assisted polishing (MAP), surface roughness, material removal rate (MRR)

## Abstract

Silicon nitride (Si_3_N_4_) is used for high-speed rotating bearings in machine tools, aircraft, and turbo pumps due to its excellent material properties such as high-temperature strength, hardness, and fracture toughness. Grinding with fixed abrasives enables high shape accuracy and high efficiency in machining brittle materials. However, it is difficult to completely remove surface damage, which limits its use in products requiring a nano surface. These defects also result in reduced reliability and shortened lifespan. Magnetic-assisted polishing (MAP) is a technology that can achieve a fine surface by using a mixture of iron powder and abrasives, but it requires a lot of time due to the low material removal rate (MRR). Therefore, this study developed a hybrid processing technology using a metal-bonded grinding wheel and a slurry with hard abrasives for the high precision of silicon nitride ceramic ball bearings. Experiments were conducted in order to compare and analyze the surface roughness and material removal rate. Through MAP, using a grinding wheel with low grit (#325), high-efficiency machining performance was confirmed with a maximum material removal rate of 1.193 mg/min. In MAP, using a grinding wheel with high grit (#2000), a nano-level surface roughness of 6.5 nm Ra was achieved.

## 1. Introduction

With the recent advancements in the aerospace and semiconductor industries, the utilization of ceramic components has increased, along with a growing interest in ultra-precision machining technology. Ceramic ball bearings are capable of operating in high-temperature and corrosive environments where traditional bearings are limited. Silicon nitride (Si_3_N_4_) is noted for its excellent mechanical properties, such as its high-temperature strength, hardness, and fracture toughness, making it suitable for high-speed rotating applications like machine tools, aircraft, and turbo pumps [1,2,3,4,5]. Because Si_3_N_4_ bearings have superior heat and wear resistance compared to steel bearings, many efforts have been made to develop high-performance silicon nitride ball bearings as a replacement for metal materials [6]. Ball bearings are classified according to their machining precision. G5 class ball bearings must have a ball size of 9.525 mm, a ball diameter variation of 0.13 μm or less, a spherical form deviation of 0.13 μm or less, a surface roughness of 0.014 μm Ra or less, and a ball lot diameter variation of 0.25 μm or less.

However, due to their high hardness and brittleness, ceramic ball bearings require a micro-level depth of cut and low cutting resistance during manufacturing, which results in a long processing time. The demand for high shape precision also increases manufacturing costs, and conventional grinding and finishing processes often result in uncertain surface defects. These defects can compromise reliability and shorten lifespan, limiting the broader applications of silicon nitride ceramics. To use ceramics in high-performance ball bearings, it is crucial to develop manufacturing technologies that meet high precision and efficiency requirements.

In 1998, H. S. Aum and colleagues developed a high-quality ceramic ball bearing processing device using magnetic polishing technology [7]. However, the issue of improving the surface roughness of processed balls remains unresolved. In 1996, N. Umehara proposed a technique known as magnetic polishing, focusing on magnetic fluid grinding for silicon nitride ceramic ball bearing finishing [8,9]. Based on the technique, a magnetic fluid grinding device using a roller-type tool was developed for ball finishing. In 1998, M. Jiang researched an alternative technology using the magnetic float polishing (MFP) process to complete silicon nitride ceramic balls for hybrid bearings [10,11]. By using soft abrasives like cerium oxide (CeO_2_), excellent surface roughness (Ra < 4 nm, Rz < 0.04 um) can be achieved while preserving the undamaged surface of the silicon nitride ceramic balls. In 1999, R. Komanduri proposed an alternative strategy to manufacture ceramic balls, which contained an initial mechanical polishing process followed by subsequent chemical mechanical action [12,13,14,15]. The magnetic fluid polishing process is effective for surface finishing silicon nitride ceramic balls used in bearings, completing the processing of balls of the same size within approximately 16 to 20 h, significantly shorter than the weeks required by traditional polishing processes. Faster polishing times and the use of non-diamond abrasives can significantly reduce the overall manufacturing costs of silicon nitride ceramic ball bearings. In 2006, N. Umehara and colleagues designed and manufactured a new device using the MFP method to finish various sizes of silicon nitride balls for hybrid bearings [16,17]. The developed MFP technology proved effective in achieving polished surfaces for silicon nitride balls used in ball bearings across a wide range of sizes.

Meanwhile, resinoid or vitrified bonding materials are mainly used in grinding wheels to reduce particle protrusion and grinding load due to abrasive particle wear. The wear and dulling of abrasive particles in grinding wheels with resinoid or vitrified bonds increase the contact area between the wheel and workpiece, leading to increased principal and shear stress, which raises processing resistance. In contrast, grinding wheels with metal bonds, manufactured using powder metallurgy, are hard and wear-resistant, facilitating the grinding of ceramics with high strength and hardness [18,19,20,21,22,23].

Grinding with fixed abrasives allows for high shape precision and efficient processing of brittle materials, but it is challenging to completely eliminate surface damage, limiting its use for structures requiring nanoscale surfaces. Magnetic polishing, which combines the magnetism of iron powder with abrasive slurry, can achieve a relatively high material removal rate (MRR) and fine surfaces, but the overall low MRR requires significant time. Therefore, a hybrid processing technology that combines grinding with metal-bonded wheels and magnetic polishing could be effectively applied to manufacture high-precision spherical shapes and obtain a high MRR. This study developed a hybrid processing technology that utilizes both magnetic polishing for fine surface roughness and grinding wheel techniques for high precision and efficiency to manufacture ceramic ball bearings under limited conditions, followed by performance evaluation experiments.

## 2. Materials and Methods

### 2.1. Experimental Setup

**Figure 1:**
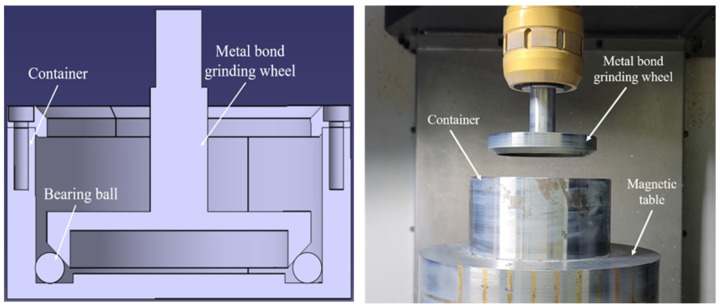
Experimental setup of magnetic-assisted polishing (MAP) using a grinding wheel.

Hybrid processing experiments on the shape of ball bearings were conducted using a vertical machining device (DNM-5700, DOOSAN, Seoul, Republic of Korea). The magnetic force required for polishing was applied using permanent magnets. The magnetic force generates a cutting force for magnetic polishing by using a mixture of iron powder and cerium oxide abrasives. Simultaneously, the metal-bonded wheel on top performs grinding process through vertical force from the depth of the cut and the rotational force from the rounding moving direction of the wheel. The magnetic polishing slurry, composed of iron powder and hard abrasives, can be used in a wet process by mixing it with oil or clean water. The grinding wheel used was a metal-bonded diamond wheel, suitable for machining high-hardness ceramic materials and minimizing wear during processing. Figure 1 shows the design and apparatus photographs of magnetic-assisted polishing (MAP) with a grinding wheel for the ball bearing. The grinding wheel was manufactured in a cup shape with a diameter of 92 mm, a thickness of 8 mm, and an inclination angle of 45°. The container was equipped with silicone pads attached to the bottom and walls to minimize damages on the balls. A permanent magnetic chuck was used to apply magnetic force to the polishing slurry, and the magnetic force measured on the pad averaged 216 G [24].

### 2.2. Experimental Methods

#### 2.2.1. Silicon Nitride Machinability Experiment

In the machining process of ceramic ball bearings, the selection of abrasive and workpiece materials is a crucial factor in obtaining a clean grinding surface, based on the correlation of material hardness, strength, and other mechanical properties. Therefore, prior to processing the ball’s shape, experiments on the machinability were conducted to choose the most efficient type of abrasive, the grit size of the grinding wheel, and the depth of cut (DOC) for processing G5 class silicon nitride balls. To effectively polish the surface of hard and brittle materials like ceramics, the Electrolytic In-Process Dressing (ELID) grinding method was applied [25,26,27,28,29]. For the experiment, a metal-bonded diamond grinding wheel and a Cubic Boron Nitride (CBN) grinding wheel were used, with grit numbers 325, 1200, and 2000 (hereafter #325, #1200, and #2000) for each type of grinding wheel. The conditions for DOC in the experiment varied according to the particle size of the grinding wheel [30,31].

#### 2.2.2. Ball Bearing Experiment

**Figure 2:**
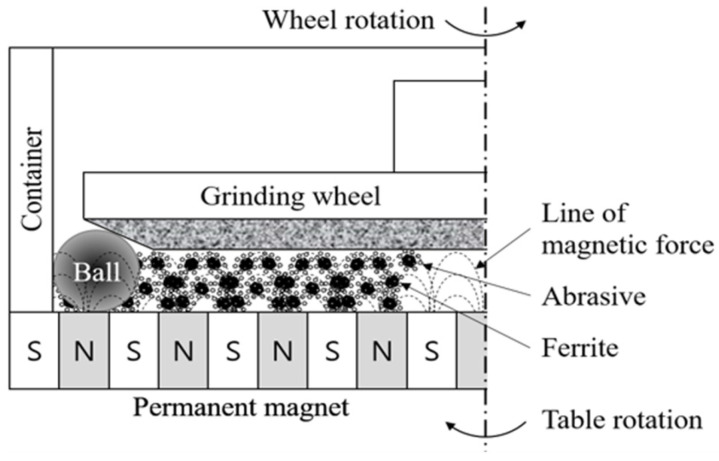
Schematic illustration of magnetic-assisted polishing (MAP) using a grinding wheel.

For the ball bearing shape processing, MAP using a metal-bonded grinding wheel was conducted, as shown in Figure 2. Based on the previous ELID experiments of flat-shaped Si_3_N_4_, efficient abrasives and grit sizes of the grinding wheel were selected, as shown in Table 1. The slurry, a wet mixture of iron powder and cerium oxide abrasives, was used for MAP, which also dressed the grinding wheel to remove chips caused by wheel loading [32,33,34,35,36].

For magnetic polishing, a container was installed on a magnetic chuck, and silicone pads were attached to the bottom and wall of the container to minimize surface defects during the ceramic ball grinding processing.

The silicon nitride balls used in the experiment were sintered with a diameter of approximately 10.5 mm, composed of Si_3_N_4_, Y_2_O_3_ (2wt%), and MgO (5wt%), and were hot-pressed (HP) under high temperature and pressure conditions at 1700 °C for 2 h. A metal-bonded diamond grinding wheel capable of achieving G5 grade surface roughness at a high grit number was used.

To reduce the total grinding time of the ball bearings, a wheel of #325 was used for high-efficiency processing experiments with MAP, and the rotational speed of the wheel was set high at 3500 rpm and 4500 rpm. The cutting feed was applied at a speed of 0.1 μm/min for 2 h in each experiment. The influence of the pressed depth (D_P_), caused by the upper grinding wheel pressing on the balls, on the surface roughness and material removal rate was evaluated when silicone pads were attached. The pressed depth was adjusted to 0 μm, 20 μm, and 25 μm for experimentation. Additionally, the influence of the rotational speed of the wheel on surface roughness and material removal rate was evaluated.

**Figure 3:**
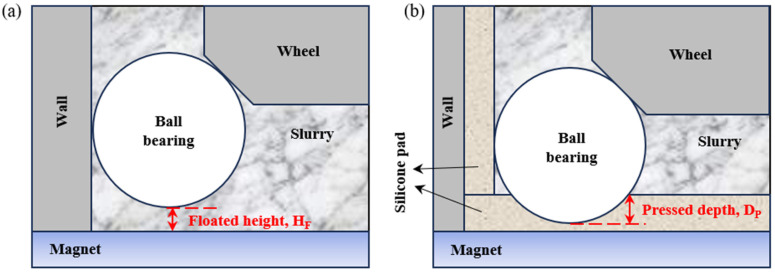
Schematic diagram of (**a**) floated height (H_F_) between ball and bottom of container in case machining center was not equipped with silicone pad and (**b**) pressed depth (D_P_) caused by upper grinding wheel with silicone pad.

To improve surface roughness after high-efficiency processing, a grinding wheel of #2000 and cerium oxide for MAP were used. To analyze the influence of the wheel’s rotational speed, experiments were conducted at 1500 rpm, 2500 rpm, and 3500 rpm, with the magnetic chuck table rotating in the opposite direction to the wheel at 20 rpm. A cutting feed was applied at a speed of 0.1 μm/min for 50 min in each experiment. When silicone pads were installed, the pressed depth was adjusted to 0 μm, 20 μm, 40 μm and 60 μm for experimentation. In the absence of a silicone pad, magnetic abrasives filled the container to create a gap between the ball and the bottom of the container, allowing the ball to be polished as it floated at a certain height in the wet slurry. The influence of the floated height (H_F_) was confirmed by adjusting the gap between the ball and the bottom of the container to 30 μm and 70 μm. Conceptual diagrams of the pressed depth and floated height are shown in Figure 3. To meet the surface roughness condition of G5 class ball bearings, additional experiments were conducted using a #2000 wheel with the rotational speed set to 300 rpm.

### 2.3. Measurement Method

The ground surface was evaluated quantitatively by measuring the surface roughness and qualitatively by analyzing surface images using a surface magnification microscope and a Field Emission Scanning Electron Microscope (FE-SEM). For measuring surface roughness, a surface profile measuring instrument (Talysurf series 2, Taylor Hobson, Leicester, UK) was used. Three different points on the surface were measured, and the average value was used for analysis. For the analysis of grinding marks and surfaces, an optical microscope (IMS345, Sometech, Seoul, Republic of Korea) and a scanning electron microscope (FE-SEM II, TESCAN, Brno, Czech Republic) were used. A micrometer (Micrometer 293-series, Mitutoyo, Aurora, IL, USA) was used for measuring the diameters of the balls to assess their shape accuracy.

To analyze the material removal rate, an electronic scale (DRAGON204/S, Mettler Toledo, Columbus, OH, USA) was used. The individual weight of the ball bearings in each experiment was measured three times, and the average value was used. The total weight was calculated by summing these average values.

## 3. Results and Discussion

### 3.1. Surface Roughness

**Figure 4:**
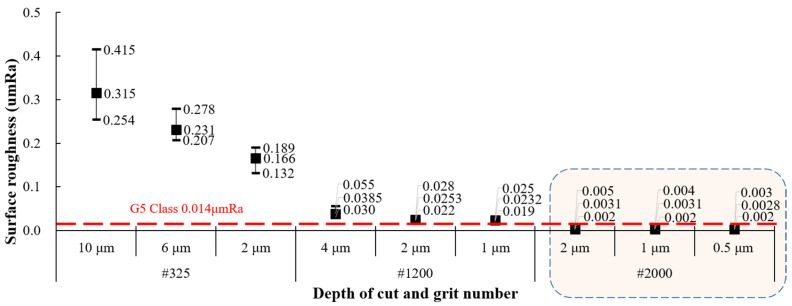
Surface roughness of the flat surface by depth of cut and grit number.

As a preliminary evaluation experiment for efficient ceramic ball processing, ELID grinding was conducted using a diamond grinding wheel and hot-pressed specimens of silicon nitride. The results of the measured surface roughness are shown in Figure 4. It was confirmed that the surface roughness and its deviation decreased as the grit size increased. For #2000, the results showed an average of 3 nm Ra across all sections, regardless of the depth of cut, which is much better than the G5 class requirement of 0.014 μm Ra. Depending on the grit size of the grinding wheel, grinding marks were observed in the same direction as the grinding for #325. However, for #2000, almost no grinding marks were observed on the surface because it was processed in ductile mode. Therefore, to conduct MAP using diamond grinding wheels for silicon nitride ceramic ball bearings, the wheels with #325 and #2000 were used for high-efficiency processing and high-precision processing, respectively.

**Figure 5:**
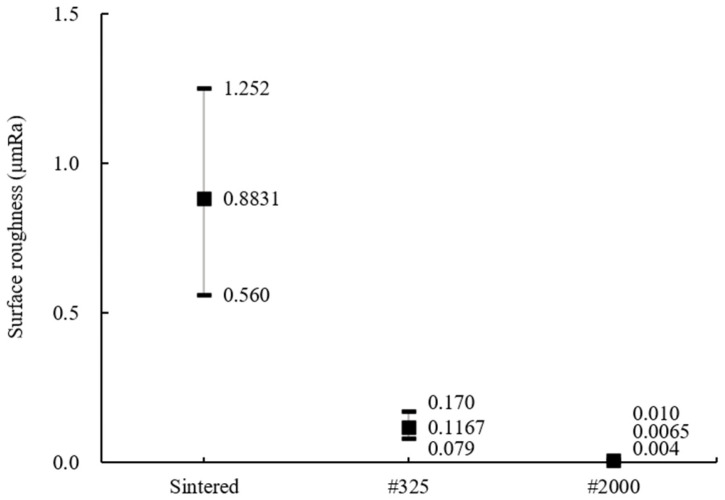
Surface roughness of the silicon nitride ball bearings by grit number.

The surface roughness of the ball bearings was examined to evaluate the ground surface by MAP using diamond grinding wheels of low and high grit size. Starting with sintered balls having an average surface roughness of 0.8831 μm Ra, the improved surface roughness measurement results are shown in Figure 5. For #325, the surface roughness decreased by almost 87% compared to the sintered ball. For #2000, average and minimum values of 6.5 nm Ra and 4 nm Ra, respectively, were obtained, showing surface roughness values far below the G5 class requirements.

**Figure 6:**
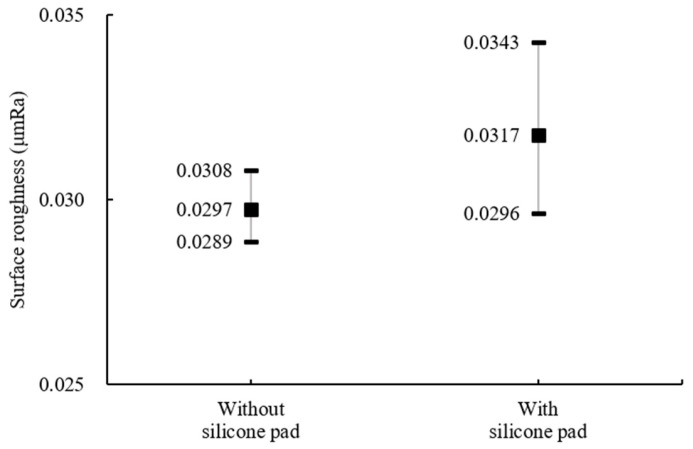
Influence of pressed depth caused by silicone pad on surface roughness (#2000).

The influence of the attached silicone pad on surface roughness, aimed at minimizing damages and defects during polishing processes, was confirmed, and the results are shown in Figure 6. When a silicone pad was used, the surface roughness increased by about 2.8%, and the deviation also increased. This is considered to be because the balls were ground by the upper grinding wheel that pressed ball bearings with direct contact, thereby increasing the influence of the wheel during processing.

**Figure 7:**
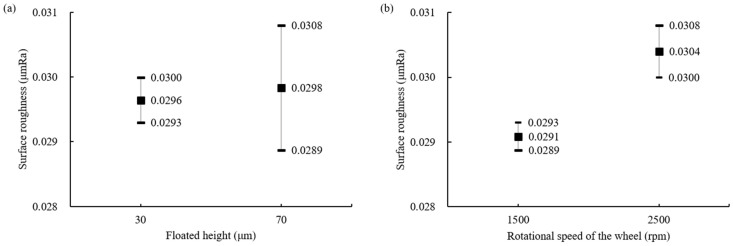
Surface roughness by (**a**) floated height and (**b**) rotational speed of the wheel (#2000, without silicone pad).

**Figure 8:**
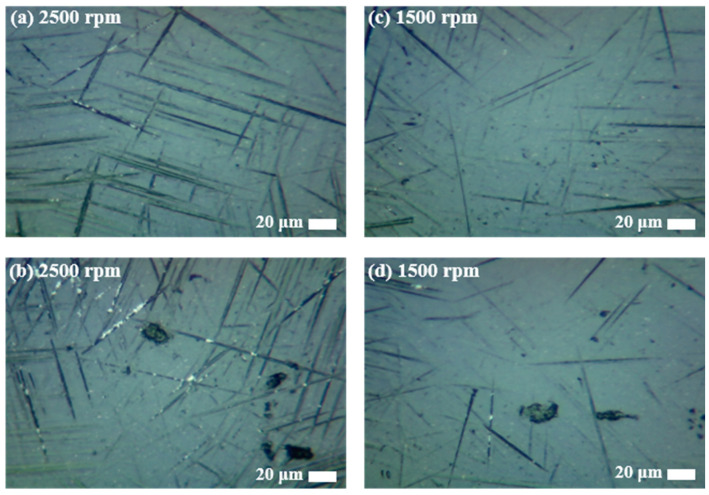
Comparison of microscopic surface images including grinding marks and spallings: (**a**) 2500 rpm with grinding marks; (**b**) 2500 rpm with spallings; (**c**) 1500 rpm with grinding marks; (**d**) 1500 rpm with spallings.

The experiments without silicone pads were conducted by adjusting the floated height (H_F_) and rotational speed of the wheel. As shown in Figure 7a, when the floated height was 70 μm and 30 μm, the difference in the average value was slight, but the deviation decreased by about three times to 6.6% and 2.3%, respectively. This suggests that when the gap between the ball and bottom of the container is small, the rotational speed of the wheel has more influence on the slurry speed. Then, it enhances the influence of the abrasives for MAP during grinding and the irregular movement of the ball decreases. Therefore, the results show relatively less deviation in surface roughness. The relationship between the rotational speed of the wheel and surface roughness is shown in Figure 7b. It was confirmed that a lower rotational speed of the wheel improved the surface condition of the ball and also reduced the deviation. The polished surface images observed under a microscope after experiments at each rotational speed of 1500 rpm and 2500 rpm confirmed the same results, as shown in Figure 8. Comparing (a) and (c), more grinding marks were observed at a wheel rotation speed of 2500 rpm. Similarly, in (b) and (d), the spalling on the surface of balls processed at 2500 rpm was deeper and more numerous.

**Figure 9:**
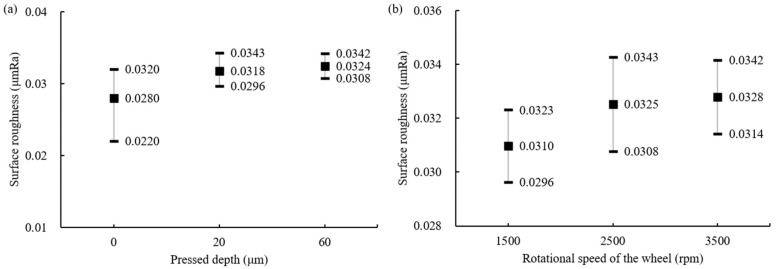
Surface roughness by (**a**) pressed depth and (**b**) rotational speed of the wheel (#2000, with silicone pad).

**Figure 10:**
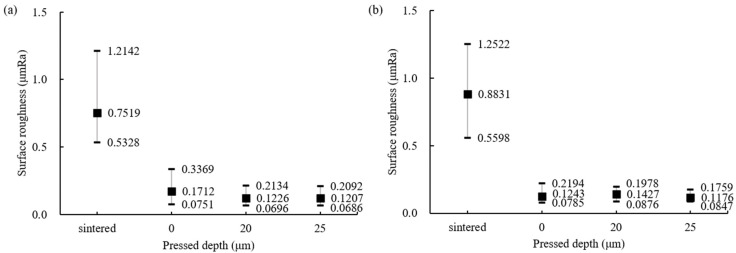
Comparison of surface roughness by pressed depth: (**a**) 3500 rpm; (**b**) 4500 rpm (#325, with silicone pad).

The effect of the pressed depth caused by a silicone pad and the rotational speed of the wheel on surface roughness was evaluated when the ball bearings were ground by MAP using #2000. As seen in Figure 9a, when the pressed depth is large, the surface roughness increases, while the deviation decreases. This indicates that the influence of the grinding wheel pressing from above becomes greater, and the irregular movement of the ball decreases as the pressed depth increases. It was also confirmed that the surface roughness gradually improves as the wheel’s rotational speed decreases, as seen in Figure 9b. In the case of #325, as shown in Figure 10, the surface roughness improved the most during the first 2 h of polishing, regardless of the wheel’s rotational speed, and then gradually improved with processing time but did not go lower than 0.1 μm Ra. This is presumed to be because most of the peaks on the surface were significantly removed during the initial processing due to the poor surface condition of the sintered balls. After the first grinding experiment of 2 h, there was a tendency for surface roughness to gradually improve. At 4500 rpm, the surface roughness was slightly but relatively lower, and the deviation decreased compared to 3500 rpm.

**Figure 11:**
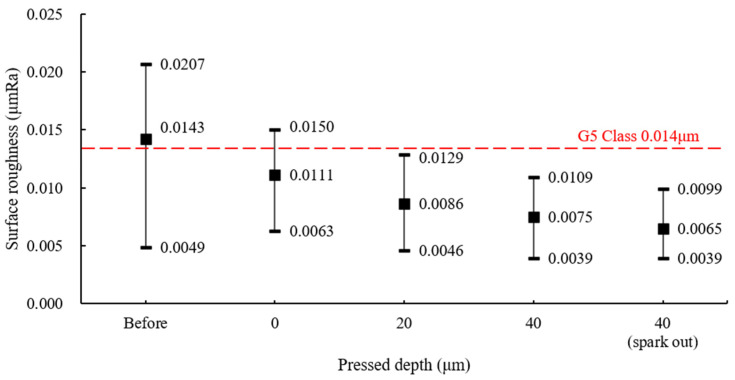
Surface roughness by pressed depth at a wheel’s rotational speed of 300 rpm (#2000).

In the experiment to achieve the surface roughness of the ball bearings meeting the G5 class requirement (<0.014 μm Ra), a wheel with #2000 was used. As in the previous results, a lower surface roughness could be obtained when the wheel’s rotational speed was lower, so the rotational speed of the wheel was reduced to 300 rpm for the experiment. Balls whose surface roughness did not meet the G5 class, with an average value of 0.0143 μm Ra and a maximum value of 0.0207 μm Ra, were processed four times by adjusting the pressed depth. The results of processing for a total of 41 h, with the pressed depth varied from 0 μm to 40 μm, are shown in Figure 11. At a pressed depth of 20 μm or more, all ground balls met the G5 class. At 40 μm, the average surface roughness was measured at 7.5 nm Ra after a total processing time of 31 h. By performing spark out processing for an additional 10 h without further increasing the pressed depth, ball bearings with an excellent average surface roughness of 6.5 nm Ra and a minimum value of 3.9 nm Ra were obtained.

### 3.2. Ball Shape Accuracy

The shape accuracy was examined by measuring the diameter of the ball bearings ground by MAP with grinding wheels. The diameters of the balls were obtained by measuring three different points along the X, Y, and Z axes. Sintered balls and balls processed with MAP using #325 showed deviations in roundness at 45 measured points, while balls processed with MAP using #2000 were measured at more points to closely approximate the actual round shape.

**Figure 12:**
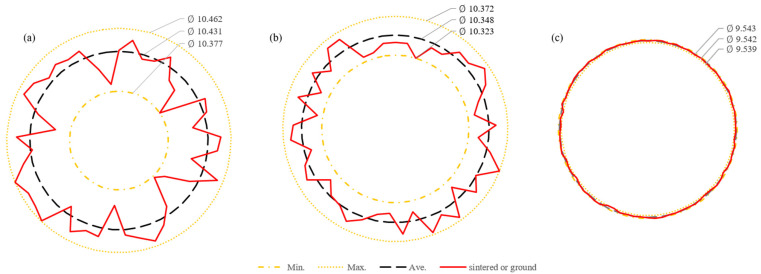
Comparison of ball roundness by the deviation in the diameters: (**a**) sintered; (**b**) ground by #325; (**c**) ground by #2000.

The results of shape accuracy depending on grit numbers are shown in Figure 12. The roundness of the sintered ball exhibited the largest diameter deviation, while the roundness of the ground balls improved with #325 and #2000, as shown in Figure 12a, Figure 12b, and Figure 12c, respectively. The diameter difference between the maximum and minimum values of the sintered balls was approximately 85 μm, while those of the ground balls by MAP using #325 and #2000 showed significant improvements, with differences of approximately 49 μm and 4 μm, respectively. When comparing the average diameter deviation of the sintered balls with that of the ground balls by MAP using #325, the shape accuracy improved by up to 44.9%, and when using #2000, it improved by approximately 88.8%.

### 3.3. Material Removal Rate

**Figure 13:**
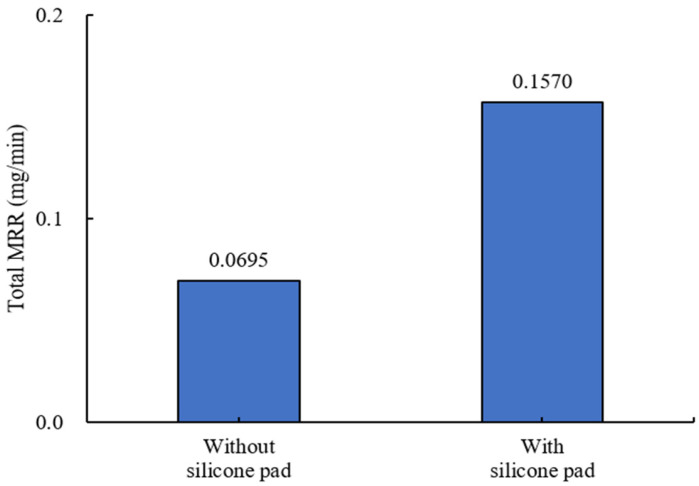
Influence of pressed depth caused by silicone pad on total material removal rate (#2000).

An experiment was conducted on the material removal rate of ball bearings processed through MAP using metal-bonded diamond wheels. As in the previous experiment, the effect of a silicone pad installed in the container on the material removal rate was examined by varying the pressed depth. As shown in Figure 13, the average total material removal rate with a silicone pad was more than twice that without a silicone pad, with differences of up to 86.3%. Therefore, it is evident that using a silicone pad during MAP can minimize damage to the balls, while adjusting the pressed depth allows for more efficient polishing.

**Figure 14:**
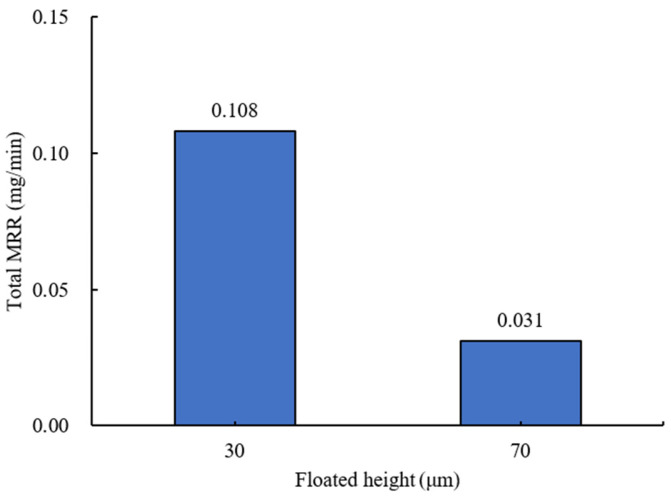
Total material removal rate by floated height (#2000, without silicone pad).

In the absence of a silicone pad, as shown in Figure 14, the floated height (H_F_) had a greater effect on the material removal rate when its value was small. The average values at 70 μm and 30 μm were 0.031 mg/min and 0.108 mg/min, respectively, showing a difference of about 3.5 times. Without a silicone pad, the ball floats in the space between the wheel and the bottom of the container, undergoing primarily magnetic polishing rather than being affected by the grinding wheels on top. When the floated height is low, the rotational speed of the wheel has a greater influence on the rotational speed of the abrasives for MAP. Thus, the slurry rotates almost at the same speed as the wheel, and the ball bearing, being heavier than the abrasives for MAP, moves slower than the slurry. This creates a greater relative speed difference between the balls and the slurry, significantly increasing the material removal rate.

**Figure 15:**
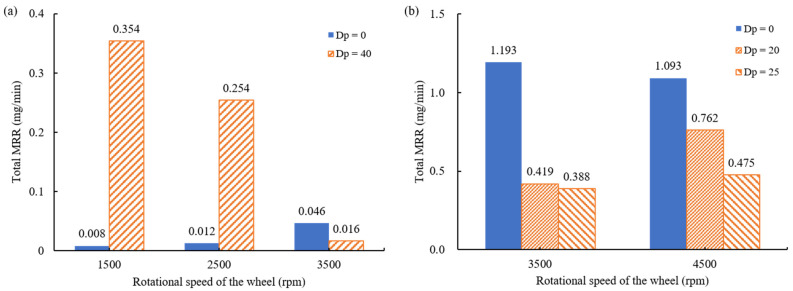
Total material removal rate by pressed depth according to the rotational speed of the wheel: (**a**) 3500 rpm and 4500 rpm using #325; (**b**) 1500 rpm, 2500 rpm, and 3500 rpm using #2000.

The effect of the pressed depth and the rotational speed of the wheel on the material removal rate was examined. For a low grit number (#325), as shown in Figure 15a, the total material removal rate decreased as the pressed depth increased and the wheel’s rotation speed decreased. The material removal rate due to the rotational speed of the wheel was greater at 4500 rpm. It is considered that a faster rotational speed of the wheel has a greater influence on the material removal rate since the friction force on the ball is larger when grinding with a low grit number. For example, at a pressed depth of 20 μm, the total material removal rates at 3500 rpm and 4500 rpm were approximately 0.419 mg/min and 0.762 mg/min, respectively, showing a difference of about twofold. When using a high grit number for the wheel, contrasting results were observed compared to those from a low grit number. As shown in Figure 15b, when increasing the pressed depth from 0 μm to 40 μm, the effect of the wheel’s rotational speed becomes greater. At 0 μm, the material removal rate increases slightly as the rotational speed of the wheel increases, whereas at 40 μm, the material removal rate increases significantly with a slower rotational speed of the wheel. Notably, at 1500 rpm, the material removal rate significantly increased from 0.008 mg/min to 0.354 mg/min. When the pressed depth was 0 μm, the results show a higher material removal rate with a faster rotational speed of the wheel because polishing by MAP had a greater effect than grinding by the wheel. However, when the pressed depth was 40 μm, the increased pressure from above caused the wheel’s rotational speed to have a greater influence on the ball’s rotational speed. This means that polishing by abrasives in the slurry is a more dominant process than grinding by the upper wheel, and the relative speed difference decreases as the wheel and ball rotate as a single unit in the container. Due to the decrease in shear stress applied to the ball, it is considered that the lowest total material removal rate occurred at 3500 rpm.

**Figure 16:**
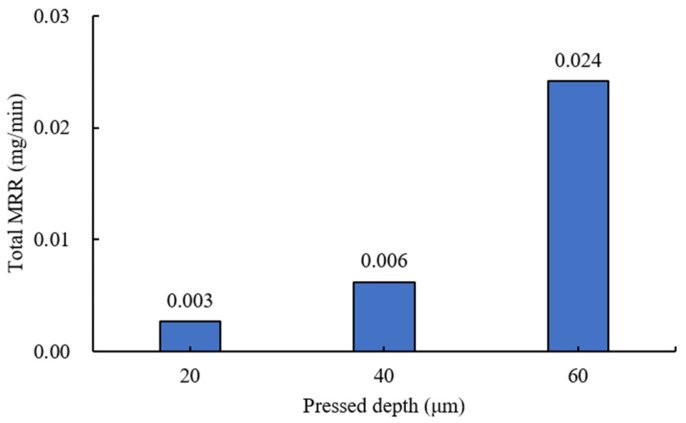
Total material removal rate by pressed depth at wheel’s rotational speed of 300 rpm (#2000, with silicone pad).

An additional experiment at 300 rpm was conducted to satisfy the G5 class requirement, and the effect of the pressed depth on the total material removal rate was examined. As shown in Figure 16, the material removal rate increases with increasing pressed depth, showing nearly a ninefold difference when the pressed depth increases from 20 μm to 60 μm. Given the previous surface roughness results, it was confirmed that more efficient grinding is achieved by MAP using a grinding wheel with a silicone pad, while minimizing surface defects on the ball bearing.

### 3.4. Analysis of Ground Surfaces

**Figure 17:**
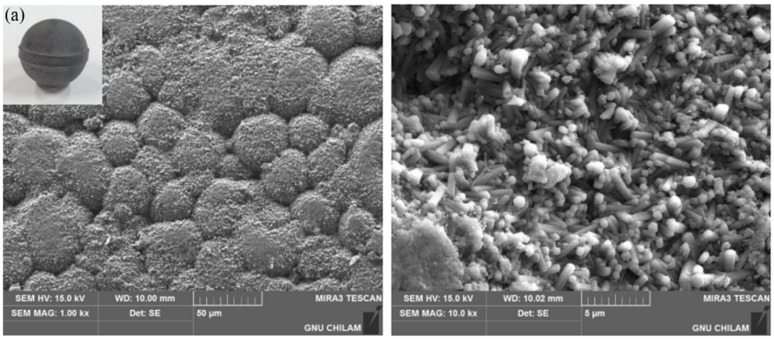

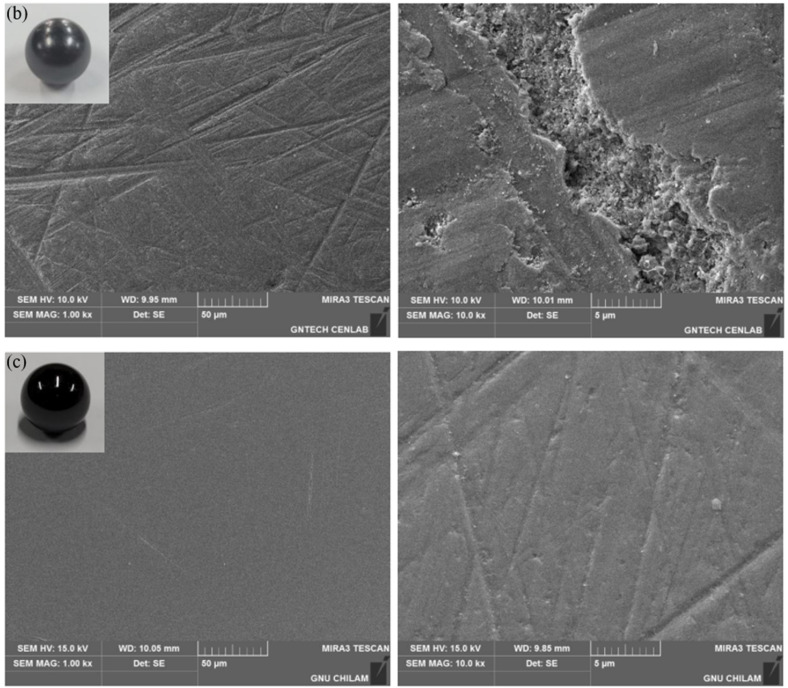
FE-SEM images of balls: (**a**) sintered; (**b**) ground by #325; (**c**) ground by #2000.

The processed surfaces of ball bearings polished through magnetic-assisted hybrid processing using metal-bonded diamond wheels were examined in this study. FE-SEM images for each ball bearing condition are shown in Figure 17. Sintered balls have bands created by sintering, and their surfaces are very rough, as seen in Figure 17a. In contrast, balls processed with MAP hybrid processing using #325 and #2000 wheels showed significantly improved surface roughness, as seen in Figure 17b and Figure 17c, respectively. In particular, using the MAP method with #2000 resulted in much better surface roughness than the G5 class. It was confirmed that using MAP with grinding wheels allows for faster rough grinding compared to conventional ball grinding methods, while also achieving excellent surface roughness.

## 4. Conclusions

By applying magnetic-assisted polishing (MAP) using metal-bonded diamond wheels, a device for manufacturing ball bearings was designed and manufactured, and its performance was evaluated. MAP experiments were conducted using a #325 wheel for rough processing and a #2000 wheel for finish processing. The effects of the floated height (H_F_) between the ball and the bottom of the container, the rotational speed of the wheel, and the pressed depth caused by the upper grinding wheel on surface roughness and material removal rate were evaluated.

- To minimize damage during the polishing process of ball bearings, a silicone pad was attached. The effect of the pressure applied to the ball by the upper wheel was examined by representing it as the pressed depth, D_P_. When there was a pressed depth, the surface roughness slightly increased, but the material removal rate became more efficient.
- As the pressed depth increased, the variation in surface roughness decreased, and better surface roughness values were obtained at a lower rotational speed of the wheel. For the material removal rate, when using #325, the total material removal rate was higher when the pressed depth was smaller. In contrast, when using #2000, the material removal rate increased significantly as the pressed depth increased.
- When a silicone pad was not installed, experiments were conducted using #2000 by adjusting the wheel’s rotational speed and the gap between the ball and the bottom of the container, represented by the floated height, H_F_. The results showed that as the floated height decreased and the rotational speed of the wheel decreased, the surface roughness values and deviation also decreased. The high-efficiency processing performance of the MAP using #325 was confirmed by the increase in the material removal rate.
- In experiments using MAP with #2000 at a very low rotational speed of the wheel, all final processed ball bearings satisfied the G5 class (<0.014 μm Ra), achieving a nano-level surface roughness with an average value of 6.5 nm Ra and a minimum value of 3.9 nm Ra.

## Figures and Tables

**Table 1:** Specific experimental conditions of magnetic-assisted polishing (MAP).

Properties	Grit Number of Grinding Wheel	
	#325	#2000
Abrasive of grinding wheel	Diamond	
Abrasives for MAP	Fe (35 μm), CeO_2_ (2.48 μm)	
Rotational speed (rpm)	3500/4500	3500/2500/1500/300
Cutting feed (μm/min)	0.1	
Processing time	2 h each	50 min. each
Floated height, H_F_ (μm)	None	30/70
Pressed depth, D_P_ (μm)	0/20/25	0/20/40/60

## Data Availability

The original contributions presented in this study are included in the article. Further inquiries can be directed to the corresponding author.
